# Clinical and Sociodemographic Determinants of Treatment Selection in Prostate Cancer: A Population-Based Study in the United States (2004–2022)

**DOI:** 10.3390/cancers18121962

**Published:** 2026-06-17

**Authors:** Manas Pustake, Atharva Railkar, Stevenson Ongsyping, Swarada Joshi, Oboseh Ogedegbe, Mohammad Arfat Ganiyani, Sumit Gaur, Jesus Gomez, Rohan Garje

**Affiliations:** 1 Department of Internal Medicine, Texas Tech University Health Sciences Center, El Paso, TX 79905, USA; atharva.railkar@ttuhsc.edu (A.R.); stevenson.ongsyping@ttuhsc.edu (S.O.); 2 Department of Internal Medicine, NRI Institute of Medical Sciences, Visakhapatnam 531162, Andhra Pradesh, India; joshiswarada51@gmail.com; 3 Department of Internal Medicine, Trinity Health Ann Arbor Hospital, Ypsilanti, MI 48197, USA; 4 Department of Medical Oncology, Miami Cancer Institute, Baptist Health South Florida, Miami, FL 33176, USA; 5 Department of Hematology and Oncology, Texas Tech University Health Sciences Center, El Paso, TX 79905, USA; 6 Department of Hematology and Oncology, University Medical Center of El Paso, El Paso, TX 79905, USA; 7 Department of Medical Oncology, Florida International University, Herbert Wertheim College of Medicine, Miami, FL 33199, USA

**Keywords:** prostate cancer, radical prostatectomy, treatment disparities, sociodemographic factors, SEER database, surgical utilization, health equity, prostate cancer management, population-based study, healthcare disparities

## Abstract

Background: Prostate cancer remains one of the most prevalent malignancies and a leading cause of cancer-related mortality among men in the United States, with more than 300,000 new cases diagnosed annually. As the burden of disease continues to rise, ensuring equitable access to effective treatment remains a critical public health priority. Unequal access to definitive surgical management among disadvantaged populations may contribute to preventable morbidity and mortality. Radical prostatectomy is a cornerstone treatment for localized disease; however, its utilization has evolved over time in response to changing screening guidelines, technological advancements, and shifting treatment paradigms. Despite these developments, disparities in access to surgical management persist. Methods: Using the Surveillance, Epidemiology, and End Results database, we conducted a retrospective cohort study evaluating factors associated with radical prostatectomy utilization among 917,194 men with localized or regional prostate cancer diagnosed between 2004 and 2022. Multivariable logistic regression assessed demographic, socioeconomic, and clinical predictors of surgical treatment. Results: Overall, 33.5% of patients underwent radical prostatectomy. Increasing age was strongly associated with lower odds of surgery (OR 0.904 per year, 95% CI 0.903–0.904, *p* < 0.001), while married patients were significantly more likely to undergo prostatectomy (OR 1.601, 95% CI 1.554–1.649, *p* < 0.001). Black patients had markedly lower odds of surgery compared with White patients (OR 0.547, 95% CI 0.539–0.555, *p* < 0.001), whereas Asian/Pacific Islander patients had slightly higher odds (OR 1.082, *p* < 0.001). More recent year of diagnosis and increasing income were associated with modest reductions in prostatectomy utilization. Conclusions: These findings demonstrate that treatment selection in prostate cancer is influenced not only by disease characteristics but also by sociodemographic factors, highlighting persistent disparities in access to definitive surgical care and the need for targeted interventions to improve equity in treatment delivery.

## Simple Summary

Prostate cancer is one of the most commonly diagnosed cancers and a leading cause of cancer-related death among men in the United States. Radical prostatectomy is a surgical procedure commonly used as definitive treatment for localized and regional prostate cancer. However, the factors influencing which patients undergo surgery are not fully understood. Using a large national cancer registry, we analyzed more than 900,000 patients with prostate cancer diagnosed between 2004 and 2022 to identify demographic, clinical, and socioeconomic predictors of radical prostatectomy utilization. Increasing age, more recent year of diagnosis, Black race, American Indian/Alaska Native race, Hispanic ethnicity, unknown race, unknown marital status, and increasing area-level income were associated with lower odds of undergoing surgery, while married status and Asian/Pacific Islander race were associated with higher surgical utilization. These findings demonstrate that non-clinical factors, including marital status, race/ethnicity, and socioeconomic context, influence treatment selection, highlighting persistent inequities in access to surgical care and underscoring the need for targeted interventions to promote equitable prostate cancer treatment.

## 1. Background and Introduction

Prostate cancer is one of the most commonly diagnosed malignancies and the second leading cause of cancer-related death among men in the United States [1,2]. It is projected to be the most commonly diagnosed cancer among men in the United States in 2026, with an estimated 333,830 new cases and 36,320 deaths, representing a significant burden on men’s health [2]. Radical prostatectomy remains a cornerstone treatment for localized prostate cancer, with treatment selection guided by risk stratification, patient preference, and life expectancy [1]. However, the landscape of prostate cancer surgical management has changed over the past two decades due to evolving screening guidelines and technological advances. Despite these advancements, persistent disparities in care delivery remain. Understanding the factors influencing surgical management is thus critical for addressing these disparities and optimizing patient outcomes.

Previous studies have examined temporal trends in radical prostatectomy utilization, but focused on a relatively narrow timespan or sample size. The use of radical prostatectomy increased across all risk groups by approximately 20% between 2004 and 2012 [3]. A similar trend was noted specifically among patients with high-risk disease, with radical prostatectomy effectively replaced external beam radiation therapy as the main avenue for treatment [3,4]. There was a shift in 2012 with the introduction of new guidelines from the United States’ Preventative Services Task Force recommendation against using prostate-specific antigen (PSA) screening, leading to a roughly 16% decrease in radical prostatectomy utilization [5]. PSA levels were found to be relatively sensitive for prostate-specific abnormalities but have poor specificity for prostate-related malignancy [5,6]. Since the implementation of these changes, different factors, such as insurance coverage and other screening methods, have influenced the implementation of radical prostatectomy [7]. More recent data suggest the stabilization of utilization patterns, possibly due to the emergence of minimally invasive techniques for radical prostatectomy that reduce the risk of complications and can result in shorter hospital stays for patients [8,9,10].

In light of such advances in care and shifting guidelines, this study aims to examine the demographic, clinical, and socioeconomic predictors of radical prostatectomy utilization using a national population-based cohort. Other large database studies have shown that racial minorities, underinsured patients, and those with otherwise low socioeconomic status are less likely to receive radical prostatectomy [3,4,11]. The most recent work indicates that race-related gaps in treatment are not explained by differences in underlying patient health, which suggests that factors influencing healthcare delivery may be involved [12,13]. Patient age and comorbidity burden also play important roles in treatment selection. Studies have shown that post-operative complications after radical prostatectomy are influenced by comorbidities more than by age alone [14]. Specifically, men with higher comorbidity burden are less likely to undergo radical prostatectomy [15,16]. Despite more research into this area, gaps remain in the understanding of factors that drive radical prostatectomy at the population level. Our goal was to examine these gaps to help reduce disparities and improve equitable access to surgical care for localized prostate cancer.

## 2. Methods

We conducted a retrospective cohort analysis using the Surveillance, Epidemiology, and End Results (SEER) database. The study included 1,259,662 male patients with a diagnosis of prostate cancer between 2004 and 2022. Of 1,259,662 total cases, 342,468 (27.2%) were excluded due to missing stage (n = 210,529), distant metastatic disease (n = 68,690), or unknown/unstaged classification (n = 63,249). The final analytic cohort included 917,194 cases with in situ, localized, or regional disease. The primary outcome examined was receipt of radical prostatectomy, defined using SEER treatment codes. We examined patient level variables, including age at diagnosis, race/ethnicity, marital status, tumor stage, treatment year, and socioeconomic factors. These variables are described in Supplementary Table S1.

A multivariable logistic regression was used to identify independent predictors of radical prostatectomy utilization. The dependent variable was receipt of radical prostatectomy (yes/no). Covariates included year of diagnosis, age at diagnosis (continuous, per year increase), race/ethnicity, median household income (inflation-adjusted to 2023 USD, 14 ordinal categories), marital status (married/common law, single, divorced, widowed, separated, and unmarried/domestic partner, and unknown).

Missing data were minimal across most variables. There were no missing values for sex, race/ethnicity, stage, or treatment variables. Age at diagnosis had 4645 missing observations (0.5%). Income had 211 cases (<0.1%) categorized as unknown/missing. Marital status included 115,438 patients (12.6%) recorded as unknown, which was retained as a separate analytic category. Overall, the dataset demonstrated high completeness, with missingness unlikely to meaningfully bias the primary analyses A complete case analysis approach was followed. All analyses were performed using SPSS (IBM Corp., version 31). Statistical significance was defined as a two-tailed *p*-value of less than 0.05.

## 3. Results

A total of 1,259,662 patients with prostate cancer were identified from the SEER database between 2004 and 2022. After excluding non-localized cancer, the final analytic cohort included 917,194 cases with in situ, localized, or regional disease. To characterize the study population and provide context for subsequent adjusted analyses, baseline demographic, clinical, and socioeconomic characteristics were first evaluated descriptively. Table 1 depicts the baseline characteristics of the cohort. The study cohort included 917,194 patients diagnosed with prostate cancer between 2004 and 2022. All patients were male. The cohort was predominantly non-Hispanic White (69.0%), followed by non-Hispanic Black (14.5%) and Hispanic (9.5%) individuals. Most patients were diagnosed with localized disease (85.8%), while 14.2% had regional-stage disease. The majority of patients did not undergo lymph node surgery (76.0%), whereas 15.6% had ≥4 lymph nodes removed. Prostatectomy was performed in 33.5% of patients. Most individuals were married at diagnosis (65.9%). Socioeconomically, 43.3% resided in areas with median household incomes between $75,000 and $99,999, and 22.5% in areas ≥$100,000. Age at diagnosis was centered in the mid-to-late 60s, consistent with the typical epidemiology of prostate cancer.

To determine whether observed differences in prostatectomy utilization persisted after adjustment for potential demographic and socioeconomic confounders, we performed a multivariable logistic regression analysis. Of 917,194 eligible cases, 912,339 (99.5%) were included in the multivariable analysis. A total of 4855 cases (0.5%) were excluded due to missing covariate data, primarily age. In the model, increasing age was strongly associated with lower odds of undergoing prostatectomy (OR 0.90 per year, 95% CI 0.903–0.904, *p* < 0.001), indicating a substantial decline in surgical utilization with advancing age. Similarly, more recent year of diagnosis was associated with a modest reduction in prostatectomy use (OR 0.99 per year, *p* < 0.001). Significant racial and ethnic disparities were observed: Black patients had markedly lower odds of receiving prostatectomy compared with White patients (OR 0.55, 95% CI 0.539–0.555, *p* < 0.001), while Asian/Pacific Islander patients had slightly higher odds (OR 1.08, *p* < 0.001). Marital status demonstrated a strong association with treatment selection, with married individuals significantly more likely to undergo prostatectomy (OR 1.60, 95% CI 1.554–1.649, *p* < 0.001), whereas those with unknown marital status had substantially lower odds (OR 0.38, *p* < 0.001). Increasing income was associated with a small but statistically significant decrease in the likelihood of prostatectomy (OR 0.98 per category increase, *p* < 0.001). Overall, the model demonstrated moderate explanatory power (Nagelkerke R^2^ = 0.222) and good classification performance, with higher specificity than sensitivity (Table 2).

## 4. Discussion

In this large SEER-based cohort of 917,194 men with in situ, localized, or regional prostate cancer, radical prostatectomy utilization was significantly associated with age, year of diagnosis, marital status, race/ethnicity, and area-level household income. These findings demonstrate that treatment selection in prostate cancer is shaped not only by clinical considerations but also by sociodemographic characteristics that may influence access to, referral for, and selection of definitive surgical management. In the adjusted model, increasing age, more recent year of diagnosis, Black race, American Indian/Alaska Native race, Hispanic ethnicity, unknown race, unknown marital status, and increasing income category were associated with lower odds of prostatectomy, whereas married status and Asian/Pacific Islander race were associated with higher odds of prostatectomy. Together, these findings highlight the multifactorial nature of treatment decision-making in prostate cancer and underscore the persistent influence of non-clinical factors on access to definitive surgical care.

The strong inverse association between increasing age and radical prostatectomy utilization (OR = 0.904) is consistent with findings from other studies Postoperative complications after radical prostatectomy are more dependent on patient comorbidities than on age alone [14]. There may be a decreasing benefit from surgery once patients are beyond the age of 70, particularly among those with lower-stage disease [16]. Our findings align with these data and with guidelines from the National Comprehensive Cancer Network, which only recommends radical prostatectomy for patients with local-ized disease, a life expectancy exceeding 10 years, and without other complex comorbid-ities [17]. However, because comorbidity burden and functional status were not available in SEER, the observed age effect may partly reflect unmeasured clinical factors that ap-propriately influence surgical candidacy.

We found that a more recent year of diagnosis was associated with lower odds of radical prostatectomy (OR = 0.99), a finding that reflects some differences in trends. While the updated guidelines in 2012 led to a reduced utilization of radical prostatectomy nationally, the increased use of surveillance for low and intermediate-risk disease has also contributed to a substantial reduction in the proportion of men undergoing surgery [5]. A recent study by Monda et al. found a 5-fold decrease in the proportion of prostatectomies with patients in a low-grade diagnosis, showing improved diagnostic pathways [18]. Thus, the observed decline in surgical odds over time is consistent with a broader shift away from routine surgery for lower-risk disease and toward more selective use of radical prostatectomy. One of the more significant findings from this study was the strong association between married status and radical prostatectomy utilization (OR = 1.60). Denberg et al. showed that marriage may be predictive of curative treatment and prostatectomy over radiotherapy in a Medicare patient cohort from the SEER database [19]. Moreover, additional studies have shown that unmarried men were less likely to receive definitive treatment, especially among unmarried Black men, and that people living with a spouse or partner were associated with a lower likelihood of care management with radiation therapy or surveillance compared to radical prostatectomy [20]. Our findings are consistent with the notion that the treatment selection pathway may be a key mechanism through which marriage confers a survival advantage [21]. Several mechanisms may underlie this association. Partners and spouses are often involved in shared decision making about treatment options with healthcare providers. Studies have shown that being married or cohabitating is associated with reduced decision-making conflict and difficulty [22]. Married patients may also benefit from support in navigating complex healthcare systems, such as scheduling, care coordination post-operatively, and facilitating access to surgical centers [23]. On the contrary, unmarried patients have significantly higher rates of watchful waiting compared to married patients, which suggests that unmarried men may lack support structures that help facilitate the pursuit of definitive surgical treatment [24]. Specifically, lower rates of marriage among Black men may signal decreased support for treatment-related decisions and caregiver support, which may be compounding the racial disparities in prostate cancer [25]. Thus, the magnitude of the effect of marital status may reflect the role of spousal support in navigating complex treatment decisions, facilitating postoperative recovery, and overcoming barriers to care [26]. The markedly lower odds among patients with unknown marital status should be interpreted cautiously, as this category may reflect incomplete documentation, social vulnerability, or other unmeasured barriers to care.

Racial and ethnic disparities were also evident. Compared with non-Hispanic White patients, non-Hispanic Black patients had markedly lower odds of undergoing radical prostatectomy (OR = 0.54). Non-Hispanic American Indian/Alaska Native patients also had lower odds of surgery (OR = 0.75), while Hispanic patients had slightly lower odds (OR = 0.97). In contrast, non-Hispanic Asian/Pacific Islander patients had slightly higher odds of prostatectomy compared with non-Hispanic White patients (OR = 1.08). Existing literature has shown that, generally, Hispanic men are less likely to receive definitive therapy compared with non-Hispanic White men [27,28,29]. The slightly lower adjusted odds among Hispanic patients in our study are directionally consistent with some prior work, although the magnitude of association was small and should be interpreted cautiously. Recent studies have shown significant variation within ethnic groups separated by country of origin [30,31]. Beyond race and ethnicity, the mechanisms driving treatment disparities are multifactorial and warrant deeper consideration. Medical mistrust represents a significant barrier, particularly among Black men, who exhibit significantly higher levels of distrust in the healthcare system compared to White men, leading to delayed care, misperceptions about disease severity, and reduced engagement in shared decision-making [32]. Studies have found that primary care practitioners may not uniformly value PSA testing for early detection or appreciate its role in reducing prostate cancer mortality among Black men, effectively functioning as gatekeepers who limit access to timely diagnosis and subsequent surgical treatment [32]. Thus, it is important to interpret our results with caution given the complexity of racial and ethnic associations with treatment selection. Moreover, our findings underscore the need for further stratified analysis in the future.

Current guidelines recommend radical prostatectomy as a potential option for patients with regional disease [17]. Moreover, other studies have shown an increase in the utilization of radical prostatectomy for locally advanced prostate cancer among patients both within and outside of the United States [33]. While our study was limited to patients within the United States, this finding is consistent with a growing role of surgery as part of treatment. The growing adoption of minimally invasive robotic surgery represents an important consideration in prostate cancer treatment, as robotic-assisted radical prostatectomy is particularly well suited for this pathology. Additionally, the use of indocyanine green fluorescence imaging has shown promise in enhancing lymphadenectomy during minimally invasive pelvic procedures [34]. The increased use of robot-assisted radical prostatectomy (RARP) further supports the surgical approach. RARP is emerging as an increasingly utilized surgical approach for localized prostate cancer with notable advantages, including reduced blood loss and shorter post-operative recovery periods [35,36,37].

These findings carry important implications for health policy and prostate cancer care delivery. The persistent association of race/ethnicity, marital status, and socioeconomic status with radical prostatectomy utilization suggests that access to definitive surgical treatment remains uneven despite advances in prostate cancer management. At the systems level, prior studies demonstrating improved cancer treatment access following Medicaid expansion suggest that continued expansion of Medicaid eligibility and reimbursement support for urologic oncology services may help reduce barriers to surgical care among low-income populations [38,39]. Additionally, adherence to Commission on Cancer quality standards emphasizing timely multidisciplinary evaluation and appropriate referral pathways may improve equitable access to definitive treatment, particularly for patients treated in lower-resource settings [40]. Given the lower rates of prostatectomy observed among Black and American Indian/Alaska Native patients, implementation of standardized referral protocols for localized and regional prostate cancer within integrated health systems may help reduce provider-level variation in treatment recommendations. Expanding patient navigation and survivorship support programs, particularly for unmarried patients who may lack caregiver assistance, could further improve completion of surgical evaluation and perioperative care. Finally, as active surveillance becomes increasingly utilized nationally, policymakers and professional societies should ensure that surveillance protocols are applied consistently across demographic groups so that evolving practice patterns do not unintentionally exacerbate existing disparities in access to curative treatment.

This study benefits from a large sample and the inclusion of multiple demographic, clinical, and socioeconomic factors within a single analysis. However, several limitations should be acknowledged. As with all registry-based studies, the SEER database does not capture certain clinically relevant variables, including comorbidity burden, insurance status, physician recommendations, treatment facility characteristics, patient preferences, and functional status, all of which may influence treatment selection. The absence of these variables may have resulted in residual confounding. For example, the observed inverse association between age and prostatectomy utilization may be partially attributable to a higher burden of comorbid illness among older patients, potentially leading to an overestimation of the independent effect of age. Similarly, the racial and socioeconomic disparities observed may be influenced by unmeasured differences in insurance coverage, access to high-volume surgical centers, referral patterns, and healthcare utilization. In addition, some degree of disease misclassification is possible, particularly among patients with incomplete staging information. Because distant metastatic disease was excluded from the analytic cohort, the present study cannot evaluate radical prostatectomy utilization among patients with metastatic prostate cancer. Finally, the retrospective observational design precludes causal inference, and the modest explanatory power of the model (Nagelkerke R^2^ = 0.222) suggests that important determinants of treatment decision-making remain unmeasured within the registry.

Future research should focus on prospective studies that incorporate detailed comorbidity profiles, functional status measures, and patient-reported treatment preferences to better characterize factors influencing surgical decision-making. Linkage of cancer registry data with insurance claims and administrative databases may provide additional insight into the roles of healthcare access, reimbursement structures, and care utilization patterns in shaping treatment disparities. Further investigation is also needed to evaluate interventions designed to reduce inequities in prostate cancer care, including patient navigation programs, multidisciplinary referral pathways, and community-based outreach initiatives. Such efforts may help identify actionable strategies to improve equitable access to definitive treatment and reduce disparities in prostate cancer outcomes.

## 5. Conclusions

In this large population-based study of 917,194 men with in situ, localized, or regional prostate cancer, radical prostatectomy utilization was significantly associated with age, year of diagnosis, marital status, race/ethnicity, and area-level socioeconomic factors. Increasing age and more recent year of diagnosis were associated with lower odds of surgery, while marital status and race/ethnicity demonstrated substantial associations with treatment selection. These findings highlight persistent sociodemographic disparities in surgical utilization and underscore the need for targeted interventions, standardized referral pathways, and patient navigation strategies to improve equitable access to definitive prostate cancer treatment.

## Figures and Tables

**Table 1:** Baseline Characteristics of Study Cohort (N = 917,194).

Characteristic	N (%)/Mean/SD/Median/IQR
Sex	
Male	917,194 (100.0)
Year of Diagnosis	
2004–2009	300,790 (32.8)
2010–2015	268,701 (29.3)
2016–2022	347,703 (37.9)
Race/Ethnicity	
White	632,888 (69.0)
Black	132,777 (14.5)
Hispanic	87,119 (9.5)
Asian/Pacific Islander	46,891 (5.1)
AI/AN	3587 (0.4)
Unknown	13,932 (1.5)
Stage at Diagnosis	
In situ	8 (0.0)
Localized	787,102 (85.8)
Regional (all)	130,084 (14.2)
Lymph Node Surgery	
None	696,900 (76.0)
1–3 nodes removed	60,820 (6.6)
≥4 nodes removed	142,763 (15.6)
Other/unknown	16,711 (1.8)
Prostatectomy	
No	609,516 (66.5)
Yes	307,678 (33.5)
Marital Status	
Married	604,729 (65.9)
Single	93,408 (10.2)
Divorced	58,368 (6.4)
Widowed	36,050 (3.9)
Other/unknown	124,639 (13.6)
Median Household Income (2023-adjusted)	
<$50,000	44,063 (4.8)
$50,000–$74,999	269,840 (29.4)
$75,000–$99,999	396,640 (43.3)
≥$100,000	206,440 (22.5)
Age at Diagnosis	
Mean (SD)/Median (IQR)	66.31 (8.7)/66 (12)

Continuous variables are presented as mean (SD) or median (IQR), as appropriate.

**Table 2:** Multivariable Logistic Regression Analysis of Factors Associated with Prostatectomy.

Variable	Category	Adjusted OR	95% CI	*p*-Value
Age (continuous)	Per year increase	0.904	0.903–0.904	<0.001
Year of diagnosis	Per year increase	0.990	0.990–0.991	<0.001
Marital status	Divorced	1.035	1.000–1.072	0.049
	Married	1.601	1.554–1.649	<0.001
	Separated	1.049	0.986–1.116	0.130
	Single	1.004	0.971–1.037	0.820
	Unknown	0.377	0.365–0.391	<0.001
	Unmarried/Partner	1.265	1.151–1.391	<0.001
Race/Ethnicity	Hispanic (all races)	0.977	0.962–0.993	0.006
	NH American Indian/Alaska Native	0.756	0.699–0.817	<0.001
	NH Asian/Pacific Islander	1.082	1.058–1.106	<0.001
	NH Black	0.547	0.539–0.555	<0.001
	NH Unknown race	0.342	0.323–0.361	<0.001
	NH White	Reference	—	—
Median household income	Per unit increase	0.979	0.977–0.981	<0.001

Adjusted odds ratios (ORs) and 95% confidence intervals (CIs) were estimated using multivariable logistic regression. Model fit was assessed using the Hosmer–Lemeshow goodness-of-fit test.

## Data Availability

The data that support the findings of this study were obtained from the Surveillance, Epidemiology, and End Results (SEER) database. SEER data are publicly available to qualified researchers upon registration and completion of a data use agreement through the National Cancer Institute SEER Program.

## Supplementary Materials

The following supporting information can be downloaded at https://www.mdpi.com/article/10.3390/cancers18121962/s1, Table S1: Definition of Covariates Included in Multivariable Logistic Regression Analysis;
