# Supplementary material for: Clinical and Sociodemographic Determinants of Treatment Selection in Prostate Cancer: A Population-Based Study in the United States (2004–2022)
Source: Cancers (Basel). 2026 Jun 17;18(12):1962. doi: 10.3390/cancers18121962 (PMC13297215; doi:10.3390/cancers18121962)
Supplement: Supplementary file 1 [file cancers-18-01962-s001.zip › cancers-4330263-supplementary.pdf]

Supplementary Table S1. Definition of Covariates Included in Multivariable Logistic Regression Analysis

Variable	Definition	Coding / Operationalization
**Outcome: Radical or Extended Radical Prostatectomy**	Binary outcome indicating whether the patient underwent Radical or Extended Radical Prostatectomy.	0 = No prostatectomy 1 = prostatectomy
**Sex**	Biological sex as recorded in the cancer registry at time of diagnosis.	Male (reference) vs Female
**Year of Diagnosis**	Calendar year in which bladder cancer was diagnosed. Modeled as a continuous variable to account for temporal trends in treatment patterns.	Continuous (per 1-year increase)
**Race/Ethnicity**	Composite variable derived from registry coding, Hispanic, whites, blacks, AI/AN. These variable captures both racial and ethnic identity as available in the dataset.	-
**Marital Status at Diagnosis**	Patient-reported marital status at the time of cancer diagnosis, reflecting social support structure and potential socioeconomic correlates. Categories are defined per registry standards.	Widowed (reference) Divorced Married Separated Single (never married) Unmarried/other Unknown
**Age (10-year intervals)**	Age at diagnosis grouped into 10-year increments to model nonlinear effects while maintaining interpretability. Reflects patient physiologic reserve and surgical candidacy.	Continuous (per 10-year increase)
**Income (Ordinal)**	Area-level socioeconomic status proxy derived from median household income, categorized into ordered groups based on registry-defined thresholds. Used as a surrogate for access to care and healthcare disparities.	Ordinal variable (increasing categories reflect higher income)
**Stage at Diagnosis (Combined Summary Stage 2004)**	Tumor stage at diagnosis based on SEER Combined Summary Stage 2004 classification. This variable captures extent of disease spread and is a primary determinant of treatment strategy.	Regional (reference) Localized Distant In situ Blank/Unknown
**Data Source / Registry Encoding Considerations**	Categorical variables were encoded using indicator (dummy) variables. Reference categories were selected based on clinical relevance and distribution. String variables exceeding software limitations were truncated prior to modeling, per SPSS processing constraints.	Indicator (dummy) coding used for all categorical covariates
